# Special Issue “Parkinson’s Disease: Genetics and Pathogenesis”

**DOI:** 10.3390/genes14030737

**Published:** 2023-03-17

**Authors:** Suzanne Lesage, Joanne Trinh

**Affiliations:** 1 Sorbonne Université, Institut du Cerveau-Paris Brain Institute, ICM, INSERM U 1127, CNRS UMR 7225, Assistance Publique-Hôpitaux de Paris (AP-HP), 75013 Paris, France; 2 Institute of Neurogenetics, University of Lübeck, 23562 Lübeck, Germany; joanne.trinh@neuro.uni-luebeck.de

Parkinson’s disease (PD) is a common and incurable neurodegenerative disease, affecting 1% of the population over the age of 65. The disease has clinical and pathological definitions through its cardinal motor manifestations and substantia nigra neuronal loss associated with intraneuronal Lewy bodies, respectively. Nevertheless, the molecular mechanisms that lead to neurodegeneration remain elusive. It is becoming increasingly apparent that genetic factors contribute to its complex pathogenesis. More than 23 loci and 13 genes, including *LRRK2*, *SNCA*, *GBA1*, *PRKN*, *PINK1*, and *PARK7*/*DJ-1*, clearly linked to inherited forms of Parkinsonism, have been identified to date. The knowledge acquired from their protein products revealed pathways of neurodegeneration that Mendelian and sporadic Parkinsonism may share. These pathways include synaptic, lysosomal, mitochondrial, and immune-mediated mechanisms of pathogenesis.

This Special Issue, “Parkinson’s Disease: Genetics and Pathogenesis”, collects 12 high-quality papers, including 7 original research articles and 5 reviews, that seek to deepen the knowledge of multiple aspects related to Parkinsonism.

Two reviews by Jia et al. [1] and Elsayed et al. [2] provide a comprehensive overview of the current knowledge of PD genetics in the genotype–phenotype relationship and associated pathophysiology with a focus on genetic testing and its current challenges and limitations. In addition, Jia et al. [1] discuss the role of heterozygous mutations in genes associated with autosomal recessive PD and the impact of digenism (i.e., dual *LRRK2* and *GBA1* mutation carriers) on the clinical outcomes. It is now recognized that critical genetic differences exist according to ethnicities and regions. Including ethnic diversity, specifically under-represented populations, in PD genetics research is essential to provide novel insights regarding the generalized genetic map of the disease. It will also improve our understanding of the disease biology, pathogenesis, and health care of PD patients. In the future, global efforts will play a key role in exploiting genomic data to identify rare genetic causes of PD or to replicate important gene discoveries. Furthermore, newer global initiatives such as the Global Parkinson’s disease program (GP2) [3] will offer diverse and expansive representation of under-represented populations from different ethnic groups and geographical regions. A third review by Kim et al. [4] covers the most common mutations in PD-related genes, such as *LRRK2*, *SNCA*, *GBA1*, *PRKN*, *PINK1*, and *PARK7*/*DJ-1*, the function of these protein products, and the consequences of their mutations on the pathophysiological mechanisms leading to PD. They emphasize further consequences of these mutations using induced pluripotent stem cells (iPSCs) for a disease-in-a-dish approach and genetic animal models.

To identify potential early predictive biomarkers in PD, Mangone et al. [5] investigated the presence of immuno-stained misfolded α-Synuclein in minor salivary gland biopsies with *substantia nigra pars compacta* (SNc) damage measured by magnetic resonance imaging. They studied 27 idiopathic PD, 16 with isolated rapid eye movement sleep disorders, a prodromal form of α-synucleinopathies, and 18 healthy controls. The authors concluded that the α-Synuclein detection in minor salivary gland biopsies lacks sensitivity and specificity and does not correlate with SNc damage. In a second original paper, Usenko et al. [6] compared the gene expression profile in monocyte-derived macrophages from four healthy controls, five PD patients, and four asymptomatic relatives carrying either heterozygous *GBA1* L444P or N370S mutations. They found dysregulated genes involved in neuronal functions, inflammation and zinc metabolism in *GBA1*-PD patients, independent of the nature of *GBA1* mutations compared to the other two groups. In particular, altered expression of *DUSP1* encoding the mitogen-activated protein kinase 1 (MKP-1) phosphatase implicated in regulating apoptosis, endoplasmic reticulum stress, cell cycle, and autophagy can be considered a potential biomarker for *GBA1*-related PD. Taking advantage of newer technologies, Pantaleo et al. [7] used whole blood transcriptome data and advanced machine learning approaches for the future selection and classification of 390 early (drug-naïve) PD patients against 189 age-matched healthy controls. The authors identified approximately 500 genes implicated in a certain number of significant functions and pathways. Some have already been linked to the pathogenesis of PD (e.g., oxidative stress, inflammation, and vesicular dysfunction) and associations between PD and diseases (e.g., diabetes mellitus or inflammatory bowel disease). The narrative review by Prasuhn and Brüggemann [8] highlights the importance of one of the known PD-associated pathways: mitochondrial dysfunction as a molecular cause in monogenic and idiopathic PD. They focus on gene therapeutic targets and challenges necessary to translate molecular findings into potential clinical applications, highlighting different treatment strategies.

Epigenetic modifications cause functional gene regulation during development, adult life, and aging and have been recently implicated in neurodegenerative diseases, such as PD. The regulation of genes responsible for monogenic forms of PD may be involved in sporadic PD. Lanoré et al. [9] reviewed the epigenetic mechanisms regulating gene expression, including DNA methylation, histone modification and epigenetic changes by non-coding RNAs. An example is *SNCA*, encoding α-Synuclein, with the understanding of its regulation being a longstanding central focus for the community working on PD. The accumulation of this protein in the Lewy bodies or neurites, the identification of mutations in the coding regions of the gene or multiplications (duplications or triplications) of the whole gene in familial PD, and the strong association of single nucleotide polymorphisms (SNPs) with sporadic PD indicate the importance of this protein in the pathogenesis of the disease. Interestingly, *SNCA* contains several transcriptionally activated histone modifications and associated potential transcription factor binding sites in the non-coding regions of the gene that strongly suggest alternative regulation pathways. Thus, studies report that DNA methylation of *SNCA* may modulate its expression, particularly hypomethylation in intron 1 of *SNCA*, which was observed in several brain regions or in peripheral tissues of sporadic PD patients and an increased *SNCA* expression. On the other hand, in post-mortem midbrain samples, an enrichment of three histone modification marks, such as H3K4me3, H3K27ac, and H3K27me3, was reported in *SNCA* regulatory regions. Finally, micro-RNAs (miRNAs), such as miR-7, miR-153, and miR-34b/c, bind to the 3′-UTR of *SNCA* mRNA, destabilizing the mRNA and reducing its levels.

Genome-wide association study (GWAS) has widened our understanding of the genetics of PD and has identified more than 90 genetic loci associated with PD [10]. Jo et al. [11] performed a GWAS on dementia in 318 PD patients with dementia, 326 PD patients without dementia, and 648 healthy controls, all of Korean origin. The data analysis led to identifying the new loci of *MUL1* associated with dementia in PD, suggesting an essential role of mitochondrial dysfunction in the development of dementia in patients with PD. Two other loci containing *ZHX2* and *ERP29* were also found to be associated with dementia in PD patients. In the original research publication, Koch et al. [12] used the development of polygenic risk scores (PRSs) to summarize the effect of genetic background on an individual’s disease risk in a single number. The authors were able to replicate the performance of the PD-PRS developed by Nalls et al. [13] in an independent dataset, suggesting that the PRS may be a meaningful research tool to investigate and adjust for the polygenic component of PD. However, this tool is not relevant for individual risk prediction.

Emerging studies revealed that expansions or intermediate repeats of simple short DNA sequences could cause or act as risk factors for different neurological diseases, including PD, depending on the number of repetitions. In the original paper by Kobo et al. [14], stratified analysis in 1106 Ashkenazi PD patients and 600 ethnically matched controls suggest that intermediate-size hexanucleotide repeats (20-60 repeats) in *c9orf72* are a risk factor for PD in individuals without common Ashkenazi Jewish founder mutations in *LRRK2*, *GBA1*, or *SMPD1* compared with those with these mutations. The authors propose a model that may drive the risk for PD by the number of repeats and the genotypes of 44 informative single nucleotide variants (SNVs) within the risk-haplotype, affecting the *c9orf72* RNA expression levels. In the second original research paper, Lüth et al. [15] established a straightforward Nanopore long-read deep sequencing workflow to quantify the hexanucleotide repeat number in the *TAF1* SINE-VNTR-Alu (SVA) insertion in patients with X-linked dystonia-Parkinsonism (XDP). In addition, the authors utilized this novel technology to investigate variations within the SVA locus other than the repeat motif and to detect CpG methylation using a Cas9-targeted approach across a large-22 kb region containing the *TAF1* SVA.

Overall, this Special Issue highlights the richness of studies bringing recent advances in our knowledge on the genetic architecture contributing to PD. This volume should be an important contribution to the field by improving our understanding of the pathophysiology and thus will help with the efforts to develop targeted therapies and personalized medicine.
